# Biomarkers for Use in Monitoring Responses of Nasopharyngeal Carcinoma Cells to Ionizing Radiation

**DOI:** 10.3390/s120708832

**Published:** 2012-06-27

**Authors:** Wei Gao, John Zenghong Li, Wai Kuen Ho, Jimmy Yuwai Chan, Thian Sze Wong

**Affiliations:** Department of Surgery, The University of Hong Kong, Queen Mary Hospital, 102 Pokfulam Road, Hong Kong, China; E-Mails: weigaoi@yahoo.com.cn (W.G.); john19850920@163.com (J.Z.L.); wkho@hkucc.hku.hk (W.K.H.); chanjyw@gmail.com (J.Y.C.)

**Keywords:** nasopharyngeal carcinoma, radiotherapy, ionizing radiation, biomarkers

## Abstract

Nasopharyngeal carcinoma (NPC) is a common head and neck cancer. The incidence rate is higher in southern China and Southeast Asia in comparison with the Western countries. Radiotherapy is the standard treatment of NPC as the cancer cells are sensitive to ionizing radiation. Radiation treatment has good local control to patients with early NPC. It is essential to monitor the response of the NPC cells to radiation treatment in advance in order to select suitable treatment choice for the patients. This review aims to discuss the potential use of biomarkers in monitoring the responsiveness of NPC cells to radiation treatment.

## 1. Introduction

### 1.1. General

Nasopharyngeal carcinoma (NPC) is a head and neck cancer that originates in the nasopharynx (the upper portion of the throat behind the nose toward skull base). NPC is a non-lymphomatous squamous cell carcinoma arising from the lateral epithelial lining of the nasopharynx. In the nasopharynx, the carcinoma cells are frequently found in the pharyngeal recess (fossa of Rosenmüller) and the eustachian tube opening in the nasopharynx [1]. According to the histological classification of World Health Organization, there are three types of NPC: WHO Type 1 NPC is keratinizing squamous cell carcinoma; Type 2 NPC is non-keratinizing carcinoma; Type 3 NPC is keratinizing undifferentiated carcinoma. Different from other head and neck cancers, the prevalence of different type of NPC varies in different geographic locations. In total, the incidence of NPC is higher in Southern China in comparison with the Western countries. In North America, 25% of the NPC are type 1 NPC. In comparison, only 2% of the NPC in southern China are type 1 NPC [1]. In southern China, type 3 NPC (undifferentiated NPC) is dominant and constitutes over 90% of the total NPC. Both ethnic background and environmental factors contribute to the risk of NPC development. Chinese emigrating to Southeast Asia or North America will remain as a high risk population. In contrast, the incidence is much lower for the Chinese born in North America [1–3]. Overall, Southern China and parts of the Artic region have the highest number of reported cases [4]. Guangdong province in China and Hong Kong have the highest incidence rate around the World, with incidences ranging from 20–22 per 100,000 men and 8–10 per 100,000 females [5].

### 1.2. Nasopharyngeal Carcinoma and Radiotherapy

The mainstay treatment for early NPC is radiation therapy, as the tumor is very sensitive to radiation. Tumor stage and tumor histology have a major influence on the treatment outcomes and are a significant prognostic factor for local or regional control of NPC. Radiation treatment is effective to control the early tumor with good prognosis. The 5-year relative survival was 65%–85% for the nonkeratinizing and undifferentiated NPC and 37% for the keratinizing NPC [6,7]. Radiation treatment, however, does not prevent the development of locoregional recurrence and distant metastasis, especially when the tumor is in the advanced stage (type III or IV). For NPC patients with advanced stages (stage III, IVA, and IVB), the combination of radiotherapy with concurrent chemotherapy (CRT) has resulted in 5-year overall survival rates of 50–70% [8]. Nonkeratinizing (WHO Type 2) or undifferentiated NPC (WHO type 3) have a better response rate to radiation treatment in comparison with the WHO Type 1 keratinizing squamous cell carcinoma [9]. In comparison with the differentiated NPC, undifferentiated NPC is more aggressive, with a higher incidence of distant metastasis [7,10]. However the response rate to treatment is better than the differentiated counterpart, resulting in a better clinical outcome. Non-keratinizing and undifferentiated NPC have a significantly better regional control rate in response to radiation treatment in comparison with the differentiated keratinizing NPC [10]. Common complications after radiation treatment include acute skin and mucosal reactions. For patients treated with radiation alone, the major skin reactions include erythema, dry desquamation and moist skin desquamation; mucosal reactions including erythema, patchy mucositis, and confluent mucositis are also observed [9].

## 2. Radiotherapy

### 2.1. Cellular Responses of Cancer Cells to Radiation

Radiation kills the cancer cells by inducing cell death following DNA damage at both the structural and functional levels [11]. The water in the cell absorbs the energy from the ionizing radiation generating reactive radical intermediate such as superoxide radicals. High contents of these reactive radical intermediates such as reactive oxygen species will cause lipid peroxidation, protein modification and DNA damage [12]. Common radiation-induced DNA damages include base oxidation, DNA double-strand breaks and DNA fragmentation that could initiate apoptosis by activating cysteine aspartyl proteases (caspase). Accumulated DNA damage will also cause cell cycle arrest and delay DNA replication and inhibit cell proliferation [13]. As discussed above, the histology type of NPC is the major determining factor for the local regional control of NPC with radiation therapy. It is suggested that the radioresistant phenotype of particular NPC reduced the therapeutic efficacy and decreased the overall survival rate [14]. In order to study the toxicity of ionizing radiation on nasopharyngeal carcinoma cells and to improve the responsiveness of nasopharyngeal carcinoma cells to radiation, cellular response and molecular markers are currently under evaluation to quantify the degree of effectiveness of ionizing radiation on the carcinoma cells.

### 2.2. Methods Employed to Study the Effects of Ionizing Radiation on Cancer Cells

**Figure 1.**
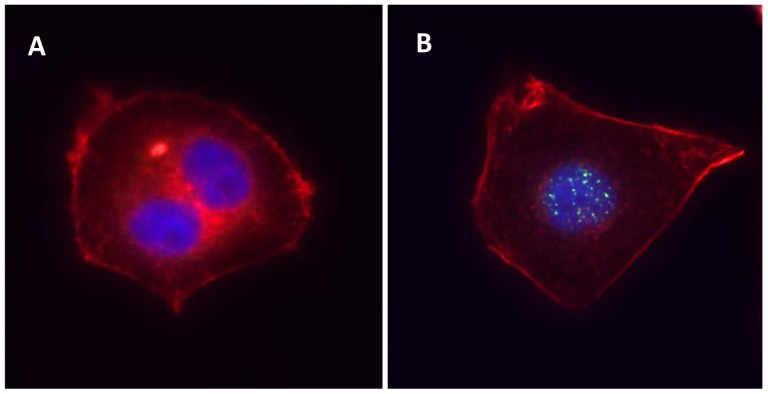
H2AX phosphorylation in undifferentiated nasopharyngeal carcinoma cell HONE1. (**A**) a dividing cell with intact nuclei without exposing to ionizing radiation; (**B**) HONE1 cell after exposing to 8 Gy ionizing radiation. The cells were fixed 24 hours after radiation, blocked and incubated with rabbit polyclonal anti-gamma H2AX (phospho S139) antibody (abcam). FITC Goat Anti-Rabbit IgG Conjugage (Invitrogen) was used to visualize phosphorylated H2AX in green. The nucleus was stained by blue-fluorescent DAPI (Invitrogen); F-actin was labeled in red with Alexa Fluor^®^ 635 phalloidin (Invitrogen).

To study the effects of ionizing radiation on carcinoma cells *in vitro*, the most commonly used method is the clonogenic assay reported by Puck and Marcus in 1956 [15]. They studied the effects of X-rays on cervical carcinoma cell line HeLa and estimated the radiation sensitivity by the capacity of the examined cells to form marcoscopic colonies under ionizing radiation. To study the effects of cell-cycle arrest after irradiation, flow cytometry is often used. As ionizing radiation could induce apoptosis by activating the the caspase family, caspase activation is employed as an index of the apoptotic response after radiation treatment. For example, caspase-3 is activated by proteolytic cleavage into two subunits (heterodimers of 17 and 12 kDa). By measuring the activated form of caspase-3, the degree of apoptotic response could be estimated [16]. Another marker of apoptotic response is the translocation of phosphatidylserine (located at the interior side of the plasma membrane) to the external surface of cell membrane. It is an early-to-intermediate response in the apoptotic process. This alteration in the plasma membrane could be monitored by Annexin V, Ca^2+^-dependent phospholipid-binding protein with high affinity to phosphatidylserine [17]. Recently, it was found that the histone H2AX (a component of the histone octomer of the nucleosomes) is subjected to heavy phosphorylation when double-strand DNA break is induced by ionizing radiation [18]. H2AX phosphorylation is a rapid response and will occur within 1 to 3 minutes after DNA damage is induced [19]. Phosphorylation of H2AX is a critical step in recruiting the DNA repair machinery [20]. The quantity of the phosphorylated H2AX could be quantified and visualized with an antibody that recognized the phosphorylation on the 139th serine residue [21]. Figure 1 demonstrates the effects of ionizing radiation on nasopharyngeal carcinoma cells *in vitro*. Further, the quantity of phosphorylated H2AX has the potential to be used as a biomarker for *in vivo* radiotherapy. To assess the relationship between radiation dose and the quantity of phosphorylated H2AX in cancer patients, Sak quantified the number of phosphorylated H2AX foci in peripheral blood lymphocytes from patients subjected to radiotherapy [22]. A linear correlation was found between them [22]. In addition to peripheral blood lymphocytes, the number of phosphorylated H2AX foci could also be quantified in paraffin-embedded biopsies from patients undergoing radiotherapy [23,24]. Qvarnström demonstrated a linear correlation between phosphorylated H2AX foci and radiation dose in skin biopsies from prostate cancer patients undergoing radiotherapy [23]. Olive found that radiation induced phosphorylated H2AX foci formation in biopsies from patients with cervical cancer [24]. However, the relationship between the number of phosphorylated H2AX foci and clinical response and outcome should be evaluated in future.

## 3. Biomarkers for Use to Monitor the Response to Ionizing Radiation

### 3.1. Epstein-Barr Virus (EBV)

EBV is a gamma herpesvirus with double-stranded genomic DNA [25]. The association between EBV and human cancer was first described in 1966 [26]. Later, it was confirmed that NPC is closely associated with EBV infection as patients usually have elevated titers of EBV specific antibodies [27]. With the use of *in situ* hybridization, EBV-encoded RNAs are detected in all the NPC tissues [28]. It has been reported that EBV-determined nuclear antigen (EBNA), early antigen (EA), EBV DNA, EBV genomic *LMP1* gene and ZEBRA protein were reliable prognosis markers for NPC patients subjected to radiotherapy. Shimakage observed that the EBNA antibody titers were increased in most sera of 36 NPC patients. The changes of the EBNA titers after ionizing radiation treatment were measured [29]. The EBNA antibody titers were reduced to normal level and maintained for a long time in 17% of patients (6/36) with good clinical prognosis after radiotherapy, while the EBNA antibody titers remained high in 17% of patients (6/36) with recurrence or metastases [29]. However, the use of EBNA antibody titers to monitor the response of radiation therapy remains controversial as it is evaluated in small cohort. In a study with 373 NPC patients (99 in Hong Kong, 120 in Tunisia and 154 in Villejuif), the values of IgG and IgA antibodies to viral capsid antigen (VCA), EA or EBNA were evaluated concurrently. It was reported that the increasing titers of IgG/EA and mainly of IgA/EA is more informative and has predictive value to disease relapse [30]. Patients with increased titer of IgG/EA exhibited higher risk of relapse in comparison with patients with stable or decreased titer (41% versus 28%) [30]. In addition, patients with increased titer of IgA/EA also displayed higher relapse rates in comparison with patients with stable or decreased titer (43% *versus* 25%) [30]. Another candidate biomarker in this regard is EBV BZLF-1 replication activator (ZEBRA) titers. 97% (85/88) NPC patients exhibited upregulated EBV BZLF-1 replication activator (ZEBRA) titers in comparison normal individuals [31]. Patients with high ZEBRA titers were found to have lower survival rate (25%) after radiation treatment in comparison with patients with low (76%) or internmediate titres (62%) [31]. Furthermore, increased titers were observed in 84.6% of patients (11/13) developing distant metastasis either in the lung or liver, while patients with remission, local recurrence or bone metastasis displayed decreased or stable titers [31]. These results demonstrated that ZEBRA titer could be used for prognosis in NPC patients upon radiotherapy.

Apart from the EBV-specific antibodies, the genomic DNA of EBV is also elevated in the peripheral blood and body fluids of NPC patients [32,33]. Serum/plasma EBV DNA was detected in most of the NPC patients. The detection frequency is much higher in NPC patients in comparison with the normal counterparts (59% *versus* 13%) in Thailand [34]. The serum/plasma EBV DNA disappeared in 92% of patients (12/13) during the early phase of treatment [34]. In a follow-up experiment, EBV DNA could not be detected in 100% of patients (32/32) with complete remission, whereas it was detectable in 60% of cases (3/5) with recurrence or partial response, indicating that EBV DNA was a valuable marker for the irradiation treatment of NPC [34]. EBV DNA was decreased after radiotherapy in 19 NPC patients with remission in Malaysia, but one recurrence case remained high EBV DNA level [35]. EBV genomic gene *LMP1* was used as a biomarker to diagnose NPC patients in Taiwan with a sensitivity of 94.7% (36/38) and a specificity of 100% (28/28) [36]. The EBV genomic *LMP1* gene was negative in 98% of patients (59/60) with overt remission, while all the patients (5/5) with local recurrence displayed positive results for *LMP1* gene, suggesting that EBV genomic *LMP1* gene was a reliable biomarker for evaluating the outcome of radiotherapy in NPC patients [36].

### 3.2. Plasma RNA Integrity

Apart from the circulating levels, the integrity of these cell-free RNA is an indicator to the clinical outcome of radiotherapy in NPC patients. Cancer patients had elevated cell-free RNA levels in their peripheral blood [37]. The RNA is mainly derived from the cancer cells with different degree of integrity in the circulation [38,39]. Wong performed an absolute quantitation of GAPDH transcript in the pre-treatment NPC plasma. In healthy individuals, there is a preponderance of the 5′ GAPDH mRNA fragment in comparison with the 3′ ends [40]. Thus, the ratio of 5′ and 3′ GAPDH transcript could be used as a non-invasive tumor maker as the RNA quality in cancer patients is usually low [41]. The RNA integrity in the circulation increased in 74% of patients (14/19) with clinical remission after radiotherapy [41].

### 3.3. Serum Metabolites

Radiation treatment will initiate specific metabolic reaction in the cancer cells. By quantifying the dynamic multi-parametric metabolic response, it is possible to identify suitable biomarkers for use to monitor the efficacy of radiation therapy [42]. Changes in endogenous metabolite levels in the body fluid involve technique used to quantity low-molecular weight compounds [42]. The low molecular weight metabolite could be detected with chromatography and mass spectrometry yielding a specific metabolite fingerprinting representing the effects of treatment. Recently, it has been shown that serum metabolites have potential to be used as markers for evaluating the clinical outcome of radiotherapy in NPC patients [43]. Kynurenine, *N*-acetylglucosaminylamine, *N*-acetylglucosamine and hydroxyphenylpyruvate levels were observed to be elevated in NPC patient sera [43]. These four metabolites were found to be reduced to the normal level and associated with rate of tumor reduction in 19 patients subjected to radiotherapy [43].

### 3.4. MicroRNA

MicroRNA are small non-protein-coding RNA, which regulate mRNA at post-transcriptional level. MicroRNA are small epigenetic regulators usually about 19–22 or 19–25 nucleotides long [44]. In comparison with the normal counterparts, nasopharyngeal carcinoma displays a differential microRNA expression pattern [45]. The underlying mechanism concerning microRNA dysregulation in nasopharyngeal carcinoma is not yet clear. It has been reported that microRNA processing machinery is upregulated during the malignant transformation of head and neck cancers [46]. MicroRNA-100 is the upstream regulator of Polo-like kinase 1 (Plk1), a serine/ threonine kinase that coordinated the mitotic process. Expression of Plk1 is upregulated in nasopharyngeal carcinoma and is linked with the decreased microRNA-100 expression [47]. Recently, another microRNA (microRNA-205) was suggested to be linked with the radioresistance of NPC cells to radiation [48]. MiR-205 expression was observed to be increased in all NPC patients after radiotherapy [48].

### 3.5. Gene Expression Patterns

Intensive studies with a wide variety of technologies have been performed to assess genes associated with radioresistance in NPC. Recently, high throughput cDNA microarrays have been employed by two studies to identify genes involved in redioresistance in NPC. Chang carried out a cDNA microarray analysis to identify genes associated with radioresistance using two resistant NPC cell lines and their corresponding parental cell lines (NPC-076 and NPC-BM1) [49]. The expressions of seven genes were observed to be increased in resistant cell lines and two of them, *gp96* and *GDF15*, were further validated by RT-PCR analysis [49]. NPC cells with silenced expression of *gp96* or *GDF15* were observed to exhibit delayed growth and reduced colonogenic survival compared to control cells upon radiation treatment [49]. Moreover, silence of *gp96* or *GDF15* resulted in elevated proportion of the cells in radiosensitive G2-M phase [49]. These results demonstrated that *gp96* and *GDF15* contributed to redioresistance in NPC cells. However, clinical data should be provided in future. In another study, Yang also employed DNA microarray to identify the genes associated with radioresistance by comparing the gene expression profiles of radiation-resistant patient biopsy specimens and radiation-sensitive patient biopsy specimens [50]. Among the 111 differentially expressed genes, the increased expression of *ZNF608* and *CSF1R* was further validated by real-time RT-PCR in 17 NPC patient specimens [50]. Pathway analysis showed that multiple pathways including cell ion homeostasis, cell proliferation, receptor protein signaling, membrane system, and humoral immune response were involved in radiation resistant in NPC [50].

Besides high throughput cDNA microarrays, proteomics was also performed to identify proteins involved in radioresistance by comparing the protein profiles of NPC cell line CNE2 and a radioresistant subclone cell line CNE2-IR [51]. Among the 34 differentially expressed proteins, the suppressed expression of 14-3-3σ and Maspin and enhanced expression of GRP78 and Mn-SOD were further validated by western blot analysis in radioresistant cell line and control cell line [51]. Moreover, the suppressed expression of 14-3-3σ and Maspin and enhanced expression of GRP78 and Mn-SOD were observed to be correlated with NPC radioresistance with a sensitivity of 90% and a specificity of 88% in the 39 radioresistant and 51 radiosensitive NPC tissues by immunohistochemistry [51]. Overexpression of 14-3-3σ partially sensitized the CNE2-IR cell to radiation [51]. These results suggested that the combination of the four proteins served as a reliable biomarker for NPC radioresistance.

In addition to high throughput cDNA microarray and proteomics, some genes associated with radiotherapy have been identified by traditional techniques, such as ELISA, immunohistochemistry, immunoelectron microscopy, in situ hybridization, western blot and real-time reverse transcription-PCR. These genes include iNOS, bcl-2, TNF-α, sIL-2R and Mn-SOD. Nitric oxide synthases (NOS) catalyzes the generation of NO, which participates in the initiation of apoptotic pathway [52]. Another protein bcl-2 is also involved in the apoptotic pathway by preventing the loss of mitochondrial membrane potential [52]. Since radiotherapy could kill cancer cells by inducing apoptosis, the roles of iNOS and bcl-2 in radioresistance are evaluated in 55 NPC patients. It was found that patients with recurrence exhibited a lower expression of iNOS with a sensitivity of 77% and a specificity of 62% and a higher bcl-2 expression with a sensitivity of 85% and a specificity of 60% [52], indicating that these two proteins were useful biomarkers for clinical prognosis of NPC patients after radiotherapy.

Tumor necrosis factor-alpha (TNF-α) is known to enhance immune effects and radiation protection by triggering release of some cytokines, such as interleukin-1, prostaglandin E_2_, γ-interferon, and tumor growth factor [53]. Blood levels of TNF-α and soluble interleukin-2 receptor (sIL-2R) were dysregulated in 58 NPC patients with different stages in comparison with healthy controls [53]. Patients with recurrence were observed to displayed upregulated expression levels of these two proteins compared with patient with complete remission, demonstrating that they were potential valuable markers for monitoring outcome of radiotherapy in NPC patients [53]. Another research also demonstrated the prognosis role of sIL-2R in NPC patients [54]. NPC patients were found to exhibited upregulated level of sIL-2R than that of controls before treatment [54]. Although patients in relapse-free and in primary relapse after radiotherapy displayed similar sIL-2R levels, 2 patients with distant metastasis were observed to have higher sIL-2R levels, indicating that sIL-2R could be used to predict distant metastasis in NPC patients after radiotherapy [54].

Manganese superoxide dismutase (Mn-SOD) catalyzes the dismutation of superoxide anion to form hydrogen peroxide. Mn-SOD could protect cells against irradiation by scavenging the reactive oxygen species generated by irradiation [55]. NPC CNE1 cells with higher expression level of Mn-SOD were more resistant to radiation than CNE2 cells [55]. Further, it was found that 48% of radioresistant tumors (11/23) exhibited Mn-SOD expression, whereas only 4% of radiosensitive tumors (2/46) displayed Mn-SOD expression [55]. Silence the expression of Mn-SOD sensitized CNE1 cells to radiation, suggesting that combination of Mn-SOD gene silencing and radiotherapy may improve clinical outcome for NPC therapy [55].

### 3.6. Positron Emission Tomography (PET)

Positron emission tomography (PET), an imaging technique based on molecular biology, can detect the physiological and biochemical aberrations of human tissues that occur in the earliest stages of diseases [56]. PET scan data can be qualitative by subjective visualization and quantitative by measuring the standardized uptake value (SUV) of tissue [57]. However, the low spatial resolution limited the use of PET. Therefore, the combination of PET and computed tomography (CT) has been developed to provide fused functional images and anatomical images [58]. ^18^F-fluorodeoxyglucose (^18^F-FDG) is widely used as PET radiotracer in oncology [57]. ^18^F-FDG-PET/CT has been applied in diagnosis, staging, prognosis and monitoring therapy response in a wide variety of cancers including lung cancer, head and neck cancer, brain cancer, lymphoma, oesophageal cancer, prostate cancer, breast cancer, colorectal cancer and pancreatic cancer [56,58].

^18^F-FDG-PET/CT has been used as a prognostic predictor in NPC patients subjected to radiotherapy [59,60]. To assess the prognostic value of ^18^F-FDG-PET/CT, Xie assessed the association of maximal standardized uptake value (SUVmax) from ^18^F-FDG-PET/CT and the therapy outcome of 62 NPC patients undergoing radiotherapy [59]. It has been found that patients with a lower SUVmax (<8.0) of tumor displayed higher 5-year overall survival and disease-free survival than those with a higher SUVmax (≥8.0) [59]. In a study including 75 NPC patients subjected to radiotherapy, Liu has also reported that NPC patients with a lower SUVmax (≤5.0) exhibited better 5-year local failure-free survival and disease-free survival than those with a higher SUVmax (>5) [60].

### 3.7. Circulating Tumor Cells

Circulating tumor cells (CTCs) are cancer cells originating from the primary tumor or metastatic sites and circulating freely in the peripheral blood of patients [61]. The metastases of solid tumors are thought to be initiated by the invasion of tumor cells into peripheral circulation [62]. CTCs have the potential to be used for prognosis and monitoring therapy response in metastatic breast cancer, prostate cancer, colorectal cancer, lung cancer, bladder cancer, renal cancer, gastric cancer and liver cancer [62,63]. Increasing evidence demonstrated that CTCs are associated with therapy outcome such as progression-free survival and overall survival [62,63]. To evaluate the CTCs level in patients with lung cancer after radiotherapy, Ge used cytokeratin 19 (CK19) as a marker of CTCs and measured the level of CK19 in peripheral blood [64]. It was found that the level of CK19 decreased after radiotherapy, indicating that CTCs can be affected by radiotherapy in lung cancer [64]. Using *CK19* as a marker of CTCs, Lin found that NPC patients with well-documented distant metastasis displayed high positive rate of *CK19* mRNA, suggesting the positive detection of CTCs in the peripheral blood of NPC patients [65]. However, the association between the level of CTCs and radiotherapy should be evaluated in NPC patients in future.

## 4. Conclusions

Although NPC cells are sensitive to radiation, the radiation dose is limited as the nasopharynx is located at the skull base in close proximity with multiple critical organs including brain stem, eyes, middle and inner ears, parotid glands and spinal cord [66]. Further, radiation treatment usually accompanies with undesirable complications including xerostomia, limb numbness, temporal lobe necrosis and neuropathy which increase the medical comorbidity and medical expenses [66]. In case of treatment failure, local recurrence is usually observed. Although the recurrent NPC patients could still receive re-irradiation, the prognosis is unfavorable, with a 5 year survival rate ranging from 8–36% [67]. Although high radiation dosage is better for local control, the prescribed dose is individualized depending on the tumor location and tumor volume. In order to monitor the efficacy of radiation treatment, the use of molecular biomarkers is beneficial as the test could be done in a non-invasive fashion using the peripheral blood or body fluids of NPC patients. The advantages and limitations of these molecular biomarkers are summarized in Table 1.

Moreover, the use of molecular biomarkers allows continuous measurement of the response rate providing more information in designing the treatment plan and selection of radiotherapy techniques. Although it is promising to use biomarkers to monitor the efficacy of the radiation therapy, several questions remain unaddressed. First, is the biomarker specific to the NPC or it is a universal marker for common radiation therapy? Most of biomarkers studied in the NPC are not evaluated in other human malignancies; second, most of the candidate biomarkers are only examined in the retrospective study and are not yet evaluated in large population size with sufficient statistical power. In addition, the predictive values of the biomarkers in relation to prognosis and treatment efficacy remain unclear; third, the biological relevance of the biomarkers to the pathology of NPC and responses of NPC cells is not yet clear. Although it seems that EBV-associated NPC is relatively sensitive to radiation, the mechanisms leading to such behavior remain unclear. Thus, further studies in large cohorts are warranted to identify accurate and reliable biomarkers for use to monitor the response of nasopharyngeal carcinoma cells to radiation therapy.

## Figures and Tables

**Table 1.** Biomarkers for monitoring responses of NPC to radiation.

**Biomarkers**	**Advantages**		**Limitations**		**References**
**EBV EBNA titers**	1.	Predict remission, recurrence or metastases	1.	Data from relative small sample size (36 patients)	[29]
	2.	Minimally invasive using serum	2.	Not useful for type I and II NPC	
	3.	Convenient			
	4.	Low-cost			

**EBV EA titers**	1.	Predict relapse	1.	limited sensitivity and specificity	[30]
	2.	Data from relative large sample size (373 patients)	2.	Not useful for type I and II NPC	
	3.	Minimally invasive using serum			
	4.	Convenient			
	5.	Low-cost			

**EBV ZEBRA titers**	1.	Predict survival rate and metastases	3.	Not useful for type I and II NPC	[31]
	2.	High sensitivity			
	3.	Data from relative large sample size (88 patients)			
	4.	Minimally invasive using serum			
	5.	Convenient			
	6.	Low-cost			

**EBV DNA**	1.	Predict remission and recurrence	4.	Not useful for type I and II NPC	[34–36]
	2.	High specificity and sensitivity			
	3.	Data from relative large sample size (65 patients)			
	4.	Minimally invasive using serum			
	5.	Convenient			
	6.	Low-cost			

**Plasma RNA Integrity**	1.	Predict remission	5.	Data from relative small sample size (19 patients)	[41]
	2.	Minimally invasive using serum			
	3.	Convenient			
	6.	Low-cost			

**Serum Metabolites**	1.	Predict remission	1.	Data from relative small sample size (19 patients)	[43]
	2.	Minimally invasive using serum	2.	Lack of data on specificity and sensitivity	
	3.	Low-cost			

**microRNA-205**	1.	Predict radioresistance	1.	Lack of data on specificity and sensitivity	[48]
	2.	Low-cost	2.	Invasive tumor tissue is needed	

***ZNF608* and *CSF1R* gene**	1.	Predict radioresistance	1.	Data from relative small sample size (17 patients)	[50]
	2.	Low-cost	2.	Lack of data on specificity and sensitivity	
			3.	Invasive tumor tissue is needed	

**Combination of 14-3-3σ, Maspin, GRP78 and Mn-SOD proteins**	1.	Predict radioresistance	1.	Combination of four proteins leads to heavy work	[51]
	2.	Data from relative large sample size (90 patients)	2.	Invasive tumor tissue is needed	
	3.	High specificity and sensitivity			
	4.	Low-cost			

**iNOS and bcl-2 proteins**	1.	Predict recurrence	1.	limited specificity	[52]
	2.	Data from relative large sample size (55 patients)	2.	Invasive tumor tissue is needed	
	3.	Low-cost			

**sIL-2R and TNF-α proteins**	1.	Predict remission and recurrence	1.	Lack of data on specificity and sensitivity	[53,54]
	2.	Data from relative large sample size (58 patients)	2.	Invasive tumor tissue is needed	
	3.	Low-cost			

**Mn-SOD protein**	1.	Predict radioresistance	1.	Limited sensitivity	[55]
	2.	Data from relative large sample size (69 patients)	3.	Invasive tumor tissue is needed	
	3.	High specificity			
	4.	Low-cost			

**PET image**	1.	Predict 5-year overall survival and disease-free survival	2.	Expensive	[59,60]
	4.	Data from relative large sample size (62 and 75 patients)			
